# Clinical characteristics and outcomes according to age in lenalidomide-treated patients with RBC transfusion-dependent lower-risk MDS and del(5q)

**DOI:** 10.1186/s13045-017-0491-2

**Published:** 2017-06-26

**Authors:** Pierre Fenaux, Aristoteles Giagounidis, Dominik Selleslag, Odile Beyne-Rauzy, Moshe Mittelman, Petra Muus, Stephen D. Nimer, Eva Hellström-Lindberg, Bayard L. Powell, Agnes Guerci-Bresler, Mikkael A. Sekeres, H. Joachim Deeg, Consuelo del Cañizo, Peter L. Greenberg, Jamile M. Shammo, Barry Skikne, Xujie Yu, Alan F. List

**Affiliations:** 1 Service d’Hématologie Séniors, Hôpital Saint-Louis, Université Paris 7, 1 Avenue Claude Vellefaux, 75475 Paris, France; 2 Marien Hospital Düsseldorf, Düsseldorf, Germany; 3 AZ St-Jan BruggeAV, Brugge, Belgium; 4 Purpan Pavillion de Medecines, Centre Hospitalier Universitaire, Toulouse, France; 5 Tel Aviv Sourasky Medical Center, Tel Aviv, Israel; 6 Radboud University Medical Centre, Nijmegen, The Netherlands; 7 Sylvester Comprehensive Cancer Center, University of Miami, Miami, FL USA; 8 Karolinska University Hospital, Stockholm, Sweden; 9 Comprehensive Cancer Center of Wake Forest University, Winston-Salem, NC USA; 10 CHU Brabois, University Center of Medicine, Vandoeuvre, France; 11 Cleveland Clinic, Cleveland, OH USA; 12 Fred Hutchinson Cancer Research Center, Seattle, WA USA; 13 Hospital Universitario de Salamanca, Salamanca, Spain; 14 Stanford Cancer Institute, Stanford, CA USA; 15 Rush University Medical Center, Chicago, IL USA; 16 Celgene Corporation, Summit, NJ USA; 17 H. Lee Moffitt Cancer Center and Research Institute, Tampa, FL USA

**Keywords:** Acute myeloid leukemia, del(5q), Lenalidomide, Myelodysplastic syndromes, Age

## Abstract

**Background:**

Particularly since the advent of lenalidomide, lower-risk myelodysplastic syndromes (MDS) patients with del(5q) have been the focus of many studies; however, the impact of age on disease characteristics and response to lenalidomide has not been analyzed.

**Methods:**

We assessed the effect of age on clinical characteristics and outcomes in 286 lenalidomide-treated MDS patients with del(5q) from two multicenter trials.

**Results:**

A total of 33.9, 34.3, and 31.8% patients were aged <65 years, ≥65 to <75 years, and ≥75 years, respectively. Age <65 years was associated with less favorable International Prognostic Scoring System (IPSS) risk and additional cytopenias at baseline versus older age groups, significantly lower cytogenetic response rates (*p* = 0.022 vs. ≥65 to <75 years; *p* = 0.047 vs. ≥75 years), and higher rates of acute myeloid leukemia (AML) progression (Gray’s test, *p* = 0.013). Lenalidomide was equally well tolerated across age groups, producing consistently high rates of red blood cell transfusion independence ≥26 weeks.

**Conclusions:**

Baseline disease characteristics and AML progression appear to be more severe in younger lower-risk MDS patients with del(5q), whereas older age does not seem to compromise the response to lenalidomide.

**Trial registration:**

ClinicalTrials.gov NCT00065156 and NCT00179621

**Electronic supplementary material:**

The online version of this article (doi:10.1186/s13045-017-0491-2) contains supplementary material, which is available to authorized users.

## Background

Myelodysplastic syndromes (MDS) occur predominantly in patients aged ≥60 years, many of whom have limited treatment options due to comorbidities, functional impairment, or poor medication tolerance [1, 2]. Studies reporting the effect of age on baseline characteristics and treatment outcomes in MDS are limited and usually involve small patient cohorts [3–5]. This study aims to assess age-related differences in baseline characteristics and outcomes with lenalidomide in red blood cell (RBC) transfusion-dependent patients with del(5q) included in two multicenter studies (MDS-003 and MDS-004; ClinicalTrials.gov NCT00065156 and NCT00179621) [6, 7].

## Methods

The methodology of these studies has been described in detail [6, 7]. Analyses were carried out on the intention-to-treat population from the MDS-003 and MDS-004 studies. Patients received lenalidomide at one of three starting doses and schedules: 5 mg/day, days 1–28 (MDS-004); 10 mg/day, days 1–21 (MDS-003 and MDS-004); or 10 mg/day, days 1–28 (MDS-003), all given in 28-day cycles. Response rates and outcomes in lenalidomide-treated patients were analyzed according to age (i.e., <65 years, ≥65 to <75 years, and ≥75 years). In MDS-004, patients in the placebo or lenalidomide 5 mg/day group without an erythroid response by week 16 or those who experienced loss of erythroid response, could cross over to the lenalidomide 5 or 10 mg/day groups, respectively. Rates of RBC transfusion independence (RBC-TI) ≥26 weeks and cytogenetic response (International Working Group 2000 criteria [8]) were compared across age groups using the Tukey test. Duration and time to RBC-TI were estimated using the Kaplan–Meier method, with differences evaluated using the Tukey test. Progression to acute myeloid leukemia (AML) was defined according to French–American–British criteria [9]. Time to AML progression was adjusted for competing risk of death from other causes using Gray’s test. Time to AML progression was assessed after lenalidomide treatment failure (defined as lack of RBC-TI ≥26 weeks or relapse after achievement of RBC-TI ≥26 weeks with lenalidomide treatment). Rates of AML progression were analyzed by time since diagnosis; between-group comparisons and overall comparisons were carried out using the Tukey test and log-rank test, respectively. Overall survival (OS) was estimated by the Kaplan–Meier method and differences evaluated using the log-rank test. OS was also assessed using a Cox proportional hazards model to adjust for differences in life expectancy. Adverse event (AE) severity was graded using the National Cancer Institute Common Terminology Criteria for Adverse Events, version 3.0, and the incidence compared across age groups using Fisher’s exact test. Granulocyte-colony stimulating factor (G-CSF) use, total lenalidomide dose received, dose reductions, and treatment discontinuation due to AEs were compared across age groups using Fisher’s exact test.

## Results and discussion

This pooled analysis included all 286 patients from the MDS-003 and MDS-004 studies who received lenalidomide from study start (Additional file 1: Figure S1). Median patient age was 69 years (range, 36–95); detailed patient characteristics according to age group are provided in Table 1. Patients were distributed approximately equally across the three age groups: 97 (33.9%), 98 (34.3%), and 91 (31.8%) patients were aged <65 years, ≥65 to <75 years, and ≥75 years, respectively. More patients aged <65 years had less favorable International Prognostic Scoring System (IPSS) risk and a higher proportion were Intermediate-1-risk than Low-risk, compared with patients in the ≥65 to <75 years (*p* = 0.035) and ≥75 years (*p* = 0.099) groups. The <65 years group had the highest proportion (48.5%) of patients with two or three cytopenias compared with the ≥65 to <75 years (38.8%) and ≥75 years (37.4%) groups. The higher IPSS categorization and additional cytopenias suggest that the disease was relatively more severe in younger patients. Although the patient population in the two trials was selected (i.e., patients fulfilled a set of inclusion criteria), this finding has not been reported previously to our knowledge.

**Table 1:** Baseline characteristics of lenalidomide-treated patients by age group

Characteristic	<65 years (*n* = 97)^a^	≥65 to <75 years (*n* = 98)	≥75 years (*n* = 91)
Age, years			
Median	59	69	79
Range	36–64	65–74	75–95
Female sex, *n* (%)	71 (73.2)^†^	56 (57.1)^‡^	72 (79.1)
Time since diagnosis, years			
Median	2.4	2.7	2.5
Range	0.2–20.7	0.2–14.7	0.1–29.2
Creatinine clearance, ml/min			
Median	117.3	88.6	55.5
Range	49.5–259.0	32.3–162.4	18.2–95.6
ECOG performance status score,^§^ *n* (%)			
0	17 (17.5)	31 (31.6)	11 (12.1)
1	23 (23.7)	22 (22.4)	30 (33.0)
2	3 (3.1)	3 (3.1)	8 (8.8)
Missing^b^	54 (55.7)	42 (42.9)	42 (46.2)
IPSS risk, *n* (%)			
Low	25 (30.5)**	36 (43.9)	37 (45.7)
Intermediate-1	53 (64.6)	37 (45.1)	39 (48.1)
Intermediate-2	4 (4.9)	6 (7.3)	5 (6.2)
High	0	3 (3.7)	0
Missing	15 (15.5)	16 (16.3)	10 (11.0)
IPSS-R risk, *n* (%)			
Very low	0	1 (1.3)	1 (1.3)
Low	32 (43.2)	30 (38.5)	41 (54.7)
Intermediate	33 (44.6)	35 (44.9)	22 (29.3)
High	8 (10.8)	8 (10.3)	8 (10.7)
Very high	1 (1.4)	4 (5.1)	3 (4.0)
Missing	23 (23.7)	20 (20.4)	16 (17.5)
Cytogenetic abnormalities, *n* (%)			
Isolated del(5q)	63 (64.9)	76 (77.6)	61 (67.0)
del(5q) plus 1 additional abnormality	22 (22.7)	11 (11.2)	17 (18.7)
del(5q) plus ≥2 additional abnormalities	8 (8.2)	5 (5.1)	11 (12.1)
Other or missing data	4 (4.1)	6 (6.1)	2 (2.2)
Number of cytopenias, *n* (%)			
1	50 (51.5)	60 (61.2)	56 (61.5)
2 or 3	47 (48.5)	38 (38.8)	34 (37.4)
Missing	0	0	1 (1.1)^c^
RBC transfusion burden, units/8 weeks			
Median	6	6	6
Range	1–15	1–25	1–12
Hemoglobin level, g/dl			
Median	7.4^‡‡^	7.8	7.9
Range	4.3–10.0	4.0–10.4	3.6–11.8
Platelet count, ×10^9^/l			
<150	23 (23.7)	32 (32.7)	29 (31.9)
≥150	74 (76.3)	66 (67.3)	62 (68.1)
ANC, ×10^9^/l			
Median	1.9	2.1	2.1
Range	0.2–21.0	0.4–10.3	0.3–20.7
Bone marrow blasts, %			
Median	3.0	4.0	2.0
Range	0.0–49.0	0.0–19.0	0.0–17.0
p53 protein overexpression in ≥1% cells, *n*/*N* (%)	11/34 (32.4)	8/22 (36.4)	11/29 (37.9)

*ANC* absolute neutrophil count, *ECOG* Eastern Cooperative Oncology Group, *IPSS* International Prognostic Scoring System, *IPSS-R* IPSS–revised, *RBC* red blood cell

^†^ *p* = 0.024 vs. ≥65 to <75 years; ^‡^ *p* = 0.002 vs. ≥75 years; ^§^ *p* = 0.001 for ≥65 to <75 years vs. ≥75 years; **p = 0.035 vs. ≥65 to <75 years; ^‡‡^ *p* = 0.006 vs. ≥65 to <75 years; *p* = 0.003 vs. ≥75 years despite intermittent/recent RBC transfusions

^a^One patient in the <65 years group was later diagnosed with acute myeloid leukemia, which had been present at the start of the study

^b^The ECOG performance status score could only be determined for patients with this information entered into the study database

^c^The number of cytopenias at baseline could not be determined for one patient due to missing ANC data

Patients in the <65 years group received a higher median number of cycles of lenalidomide than those in the ≥65 to <75 years (17 vs. 14; *p* = 0.130) and ≥75 years groups (17 vs. 10; *p* = 0.002; Table 2). The median total lenalidomide dose received was significantly higher in the <65 years group (2540.0 mg [range, 65.0–13,730.0]) than in the ≥65 to <75 years (1507.5 mg [range, 30.0–10,295.0]; *p* = 0.045) and ≥75 years groups (1070.0 mg [range, 50.0–10,980.0]; *p* = 0.001; Table 2).

**Table 2:** Lenalidomide treatment, rates of dose reduction, and discontinuation by age group

Lenalidomide	<65 years (*n* = 97)^a^	≥65 to <75 years (*n* = 98)	≥75 years (*n* = 91)
Total dose, mg			
Median	2540.0^†^	1507.5	1070.0
Range	65.0–13,730.0	30.0–10,295.0	50.0–10,980.0
Number of cycles			
Median	17.0^‡^	14.0	10.0
Range	1.0–63.0	1.0–64.0	1.0–62.0
Dose reductions, *n* (%)			
Neutropenia	33 (34.0)	31 (31.6)	23 (25.3)
Thrombocytopenia	13 (13.4)^§^	20 (20.4)	27 (29.7)
Neutropenia and thrombocytopenia	6 (6.2)	8 (8.2)	9 (9.9)
Treatment discontinuation, *n* (%)			
Any AEs	8 (8.2)^¶^	17 (17.3)	18 (19.8)
Lack of therapeutic effect	14 (14.4)	12 (12.2)	10 (11.0)
Death	2 (2.1)	4 (4.1)	8 (8.8)

*AE* adverse event

^†^ *p* = 0.001 vs. ≥75 years; *p* = 0.045 vs. ≥65 to <75 years; ^‡^ *p* = 0.002 vs. ≥75 years; ^§^ *p* = 0.008 vs. ≥75 years; ^¶^ *p* = 0.033 vs. ≥75 years

^a^One patient in the <65 years group was later diagnosed with acute myeloid leukemia, which had been present at the start of the study

Rates of RBC-TI ≥26 weeks did not differ among the age groups, nor did the median time to onset of response and duration of response (Table 3). Cytogenetic response rates (major + minor responses) were significantly higher in the ≥65 to <75 years (65.5%; *p* = 0.022) and ≥75 years groups (63.5%; *p* = 0.047) than in the <65 years group (45.1%; Table 3). The observation that younger patients had lower cytogenetic response rates despite a higher total dose is in contrast to the previously established relationship between lenalidomide dose and cytogenetic response [7, 10]. The lower response rate may have resulted from the relatively more severe disease characteristics in younger patients. In a previous analysis of p53 status in MDS-004, *TP53* mutation and high p53 protein expression were identified as significant factors for reduced cytogenetic response in a subset of patients with biopsies available [11]. In the current study, however, no differences in p53 protein overexpression were observed across the three age groups (Table 1), whereas *TP53* mutation status could not be assessed.

**Table 3:** RBC-TI ≥26 weeks and cytogenetic response by age group in patients treated with lenalidomide

Response	<65 years (*n* = 97)^a^	≥65 to <75 years (*n* = 98)	≥75 years (*n* = 91)
RBC-TI ≥26 weeks, *n* (%)	54 (55.7)	53 (54.1)	41 (45.1)
Median time to RBC-TI ≥26 weeks, weeks (range)^b^	2.48 (1.49–NE)	2.48 (1.36–NE)	7.80 (2.15–NE)
Median duration of RBC-TI ≥26 weeks, years (range)^b^	4.60 (2.31–NE)	3.19 (1.74–NE)	2.19 (1.20–NE)
Cytogenetic response, *n*/*N* (%)			
Major + minor	32/71 (45.1)^‡^	38/58 (65.5)	33/52 (63.5)

*NE* not estimable, *RBC-TI* red blood cell transfusion independence

^‡^ *p* = 0.022 vs. ≥65 to <75 years; *p* = 0.047 vs. ≥75 years

^a^One patient in the <65 years group was later diagnosed with acute myeloid leukemia, which had been present at the start of the study

^b^Responding patients only

Lenalidomide was relatively well tolerated in all three age groups, with low rates of treatment discontinuation. More patients discontinued due to AEs in the ≥75 years group compared with the <65 years group (19.8 vs. 8.2%; *p* = 0.033; Table 2). The most common grade 3–4 AEs were neutropenia and thrombocytopenia (Table 4). The incidence of grade 3–4 thrombocytopenia was significantly lower in patients aged <65 years than in patients aged ≥65 to <75 years (*p* = 0.004) and ≥75 years (*p* = 0.019). However, the incidence of grade 3–4 neutropenia was significantly lower in patients aged ≥75 years than in patients aged ≥65 to <75 years (*p* = 0.041). Dose reductions due to thrombocytopenia were more common in the ≥75 years group compared with the <65 years group (*p* = 0.008; Table 2).

**Table 4:** Grade 3−4 AEs by age group reported in ≥5% of patients

AE, *n* (%)	<65 years (*n* = 97)^a^	≥65 to <75 years (*n* = 98)	≥75 years (*n* = 91)
Patients with ≥1 AE	91 (93.8)	95 (96.9)	89 (97.8)
Neutropenia	73 (75.3)	75 (76.5)^†^	57 (62.6)
Thrombocytopenia	37 (38.1)^‡^	58 (59.2)	51 (56.0)
Anemia	9 (9.3)	12 (12.2)	14 (15.4)
Leukopenia	9 (9.3)	9 (9.2)	15 (16.5)
Pneumonia	4 (4.1)	10 (10.2)	9 (9.9)
Fatigue	1 (1.0)^§^	8 (8.2)	3 (3.3)
Dyspnea	2 (2.1)	4 (4.1)	7 (7.7)
Febrile neutropenia	7 (7.2)	5 (5.1)	3 (3.3)
Deep vein thrombosis	2 (2.1)	7 (7.1)	3 (3.3)
Diarrhea	6 (6.2)	7 (7.1)	5 (5.5)
Hypokalemia	4 (4.1)	1 (1.0)	6 (6.6)
Cardiac failure congestive	0^¶^	2 (2.0)	5 (5.5)
Fall	0^¶^	1 (1.0)	5 (5.5)
Alanine aminotransferase increased	5 (5.2)	2 (2.0)	2 (2.2)
Rash	4 (4.1)	5 (5.1)	4 (4.4)

*AE* adverse event

^†^ *p* = 0.041 vs. ≥75 years; ^‡^ *p* = 0.004 vs. ≥65 to <75 years; *p* = 0.019 vs. ≥75 years; ^§^ *p* = 0.035 vs. ≥65 to <75 years; ^¶^ *p* = 0.025 vs. ≥75 years

^a^One patient in the <65 years group was later diagnosed with acute myeloid leukemia, which had been present at the start of the study

G-CSF prophylaxis for neutropenia did not differ significantly across the age groups (data not shown). The lower rates of neutropenia in the ≥75 years group may reflect the reduced total dose of lenalidomide in this age group rather than variations in G-CSF use. Although grade 3–4 neutropenia occurred less frequently in patients aged ≥75 years, infectious episodes were more common (data not shown), a disparity possibly related to the known natural deterioration of the immune response in older individuals [12]. Although thrombocytopenia was one of the most common AEs with lenalidomide, the incidence of grade 3–4 bleeding events remained low across all age groups (data not shown).

**Fig. 1:**
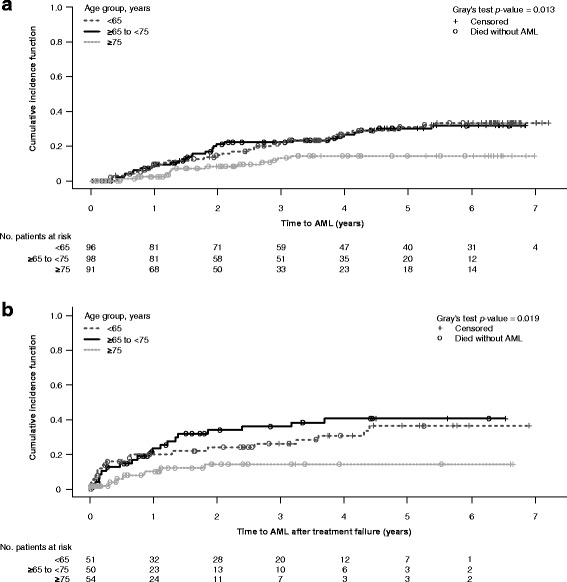
AML progression by age group in lenalidomide-treated patients (**a**) or after treatment failure (**b**). There are 52 patients in the <65 years age group, but 1 patient died on day 0 and has been excluded from the analyses. *AML* acute myeloid leukemia

The cumulative incidence of AML was significantly lower in the ≥75 years group than in the ≥65 to <75 years (*p* = 0.010) and <65 years age groups (*p* = 0.006; Fig. 1a). Similarly, the incidence of AML after treatment failure was significantly lower in the ≥75 years group than in the ≥65 to <75 years (*p* = 0.006) and <65 years groups (*p* = 0.022; Fig. 1b). To assess whether this difference could be attributed to differences in duration of MDS between age groups, rates of AML progression were analyzed according to time since diagnosis. Rates of AML did not differ significantly between patients with a median duration of MDS ≤1.5 years, >1.5 to ≤3.8 years, and >3.8 years (*p* = 0.598; Additional file 2: Table S1).

These findings are consistent with previous long-term data reported from the MDS-003 study, where achievement of cytogenetic response was associated with a lower risk of AML progression in patients with del(5q) treated with lenalidomide [13]. Although none of the individual IPSS variables differed significantly between the age groups (Table 1), a higher proportion of patients aged <65 years were classified as Intermediate-1-risk, which may explain their increased risk of AML progression. Higher IPSS categorization and presence of two or three cytopenias at baseline were associated with an increased risk of AML progression in a multivariate model based on data from the MDS-003 study [13]. In a separate analysis of untreated patients with lower-risk MDS, higher rates of AML progression were also seen in patients aged ≤65 years, with 2-year rates of 6.7 versus 3.0% in patients aged >65 years [14]. These data suggest that younger patients may have an increased risk of AML progression regardless of treatment, which is likely related to the higher-risk disease features of this patient group.

**Fig. 2:**
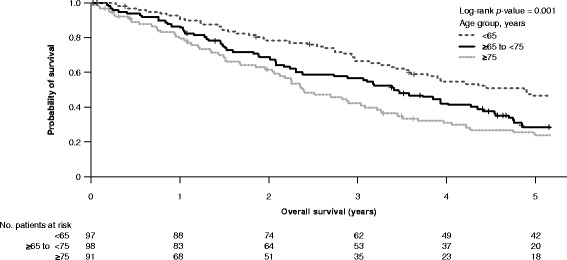
Overall survival in lenalidomide-treated patients by age group

Median OS was 4.87 years (95% confidence interval [CI], 3.58–not estimable), 3.46 years (95% CI, 2.31–4.27), and 2.40 years (95% CI, 2.04–3.22) in the <65 years, ≥65 to <75 years, and ≥75 years groups, respectively (*p =* 0.001; Fig. 2). OS was not significantly different between age groups when adjusted for life expectancy based on the overall population or when analyzed separately according to sex (data not shown). This finding suggests that the shorter OS in the ≥75 years and ≥65 to <75 years patient groups likely reflects a higher death rate due to causes other than AML progression. Concomitant disorders are indeed common in this aged population; in particular, infection and the presence of anemia were more frequent in elderly patients. Whereas younger age may be a favorable factor for survival with lenalidomide, the more severe disease characteristics seen in patients aged <65 years suggest that this group may benefit from treatment options other than lenalidomide, followed by allogeneic stem cell transplantation in case of primary or secondary lenalidomide treatment failure.

Lower creatinine clearance rates with increasing age (Table 1) result in reduced lenalidomide elimination, ultimately increasing exposure to the drug [15, 16]. It is possible that differences in drug metabolism and activity at the cellular level may have accounted for some of the apparent age-related differences observed in efficacy outcomes.

These results should be interpreted with caution given certain limitations of our analysis. For example, some mutations such as the *TP53* gene mutation and possibly mutations of other genes (i.e., *CSNK1A1*, *ASXL1*, *TET2*) are associated with a poor prognosis in patients with lower-risk MDS with del(5q) [2, 17]. Mutation data were not collected in the MDS-003 and MDS-004 studies, and therefore, it could not be determined if certain mutations were more frequent in the <65 years age group. Of note, however, p53 protein overexpression, a validated surrogate marker of *TP53* gene mutation, was not influenced by age in the present study.

## Conclusions

In summary, age <65 years was associated with poorer disease characteristics at baseline, lower cytogenetic response rates, and higher rates of AML progression than in the older age groups. These findings suggest that resistance to lenalidomide, or relapse after an initial response, may require prompt consideration of allogeneic stem cell transplantation in younger adult patients with lower-risk del(5q) MDS. On the contrary, lenalidomide was equally well tolerated across all age groups, producing consistently high rates of RBC-TI ≥26 weeks, and older age did not appear to compromise response to lenalidomide treatment.

## Additional files

Additional file 1: Figure S1. Study populations in the MDS-003 and MDS-004 studies. Gray shaded boxes: age groups in the present analysis. LEN, lenalidomide. (DOCX 39 kb)

Additional file 2: Table S1. Cumulative rates of AML progression by age group in lenalidomide-treated patients. AML, acute myeloid leukemia; MDS, myelodysplastic syndromes. (DOCX 14 kb)

## Abbreviations

AE: Adverse event
AML: Acute myeloid leukemia
ANC: Absolute neutrophil count
ECOG: Eastern Cooperative Oncology Group
G-CSF: Granulocyte-colony stimulating factor
IPSS: International Prognostic Scoring System
IPSS-R: International Prognostic Scoring System–revised
MDS: Myelodysplastic syndromes
NE: Not estimable
OS: Overall survival
RBC: Red blood cells
RBC-TI: Red blood cell transfusion independence
