# Supplementary material for: Clinical characteristics and outcomes according to age in lenalidomide-treated patients with RBC transfusion-dependent lower-risk MDS and del(5q)
Source: J Hematol Oncol. 2017 Jun 26;10:131. doi: 10.1186/s13045-017-0491-2 (PMC5485496; doi:10.1186/s13045-017-0491-2)

**Fig. S1** Study populations in the MDS-003 and MDS-004 studies. Grey shaded boxes: age groups in the present analysis. *LEN*, lenalidomide


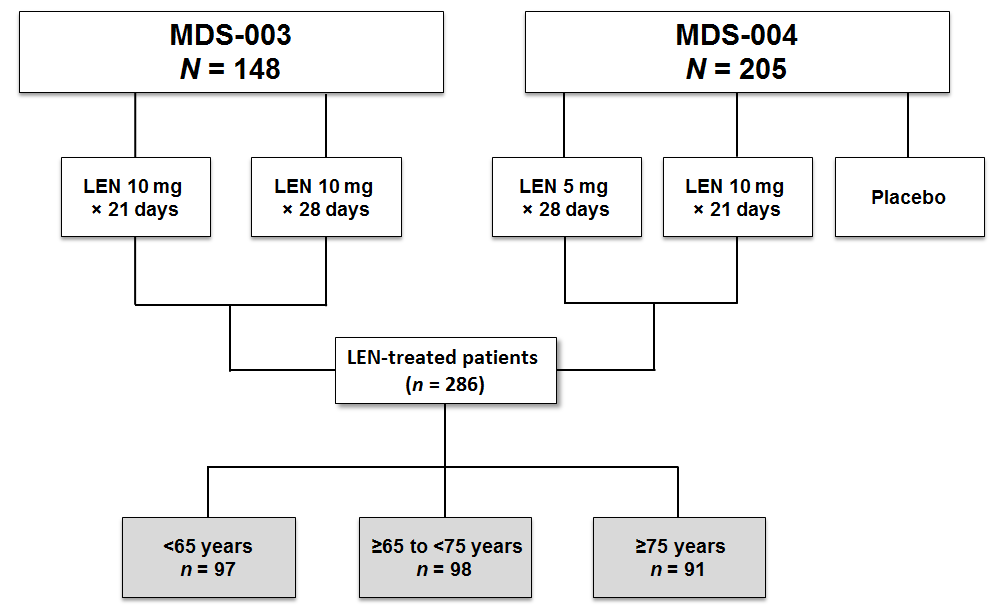

Supplement: Supplementary file 1 — Study populations in the MDS-003 and MDS-004 studies. Gray shaded boxes: age groups in the present analysis. LEN, lenalidomide. (DOCX 39 kb) [file 13045_2017_491_MOESM1_ESM.docx]
