# Supplementary material for: Clinical characteristics and outcomes according to age in lenalidomide-treated patients with RBC transfusion-dependent lower-risk MDS and del(5q)
Source: J Hematol Oncol. 2017 Jun 26;10:131. doi: 10.1186/s13045-017-0491-2 (PMC5485496; doi:10.1186/s13045-017-0491-2)
Supplement: Supplementary file 2 — Cumulative rates of AML progression by age group in lenalidomide-treated patients. AML, acute myeloid leukemia; MDS, myelodysplastic syndromes. (DOCX 14 kb) [file 13045_2017_491_MOESM2_ESM.docx]

**Table S1** Cumulative rates of AML progression by age group in lenalidomide-treated patients. *AML*, acute myeloid leukemia; *MDS*, myelodysplastic syndromes

| Cumulative rates of AML, % | Duration of MDS | | |
| --- | --- | --- | --- |
|  | ≤1.5 years  (*n* = 97)* | >1.5 to ≤3.8 years  (*n* = 89)† | >3.8 years  (*n* = 99) |
| 1-year | 5.7 | 9.9 | 6.6 |
| 2-years | 10.9 | 22.4 | 18.0 |
| 3-years | 16.6 | 28.7 | 24.5 |
| 4-years | 26.9 | 30.7 | 28.4 |
| 5-years | 33.2 | 37.2 | 30.5 |

**p* = 0.6659 vs. >1.5 to ≤3.8 years; *p* = 1.000 vs. >3.8 years

†*p* = 0.6545 vs. >3.8 years
